# High prevalence of CXCR4 usage among treatment-naive CRF01_AE and CRF51_01B-infected HIV-1 subjects in Singapore

**DOI:** 10.1186/1471-2334-13-90

**Published:** 2013-02-19

**Authors:** Kah Ying Ng, Kuan Kiat Chew, Palvinder Kaur, Joe Yap Kwan, Wei Xin Khong, Li Lin, Arlene Chua, Mei Ting Tan, Thomas C Quinn, Oliver Laeyendecker, Yee Sin Leo, Oon Tek Ng

**Affiliations:** 1 Institute of Infectious Disease and Epidemiology, Communicable Disease Centre, Tan Tock Seng Hospital, Singapore 308433, Singapore; 2 Department of Infectious Disease, Jurong General Hospital, Singapore, 159964, Singapore; 3 Johns Hopkins School of Medicine, Baltimore, MD, USA; 4 Laboratory of Immunoregulation, Division of Intramural Research, National Institute of Allergy and Infectious Disease, National Institutes of Health, Baltimore, MD, USA

**Keywords:** CXCR4 usage, HIV-1, treatment-naïve

## Abstract

**Background:**

Recent studies suggest HIV-1 inter-subtype differences in co-receptor usage. We examined the correlation between HIV-1 subtype and co-receptor usage among treatment-naïve HIV-1 subjects in Singapore. Additionally, we investigated whether the subtype co-receptor association was influenced by stage of infection.

**Methods:**

V3 sequences of HIV-1 envelope protein gp120 were obtained from 110 HIV treatment-naïve patients and genotypic co-receptor tropism determination was performed using Geno2pheno. Two false-positive rate (FPR) cut-offs, 10% and 5.75% were selected for tropism testing.

**Results:**

Subtype assignment of viral strains from 110 HIV-infected individuals based on partial sequencing of HIV-1 *pol, gp120* and *gp41* were as follows: 27 subtype B, 64 CRF01_AE, 10 CRF51_01B, and 9 other subtypes. At FPR=10%, 10 (100%) CRF51_01B-infected subjects and 26 (40.6%) CRF01_AE-infected subjects had CXCR4-using virus, compared to 7 (25.9%) subtype B subjects and 1 (11.1%) CRF33_01B-infected subject (*P* < 0.001). At FPR=5.75%, 10 (100%) CRF51_01B-infected subjects and 20 (31.3%) CRF01_AE-infected subjects had CXCR4-using virus, compared to 4 (14.8%) subtype B and 1 (11.1%) CRF33_01B-infected subjects (*P* < 0.001). Among those with evidence of seroconversion within 2 years prior to study enrolment, 100% of CRF51_01B-infected subjects had CXCR4-using virus, independent of Geno2pheno FPR.

**Conclusion:**

CRF51_01B and CRF01_AE-infected individuals have higher prevalence of CXCR4-usage compared to subtype B infected individuals. Further studies examining these differences could help optimise the use of CCR5-antagonist in populations with these subtypes, and increase our understanding of HIV-1 biology.

## Background

HIV-1 infection of immune cells involves the interaction of the *env* glycoprotein with CD4 and co-receptors. Co-receptor usage for viral entry can be divided into CCR5 (R5), CXCR4 (X4) and dual/mixed (DM) virus [1]. DM virus is able to utilize both the CCR5 and CXCR4-co-receptors. Infection with CXCR4-using (CXCR4 or DM) virus precludes therapeutic benefit from CCR5-antagonist [2]. Additionally, co-receptor switching from CCR5 to CXCR4-using virus is associated with increased rates of immunologic decline and late stage HIV disease [3,4].

Inter-subtype differences in co-receptor usage have been described in Western and African settings. In a study of 539 recently diagnosed anti-retroviral naïve HIV-1 infected individuals in Belgium, CRF01_AE infection was associated with significantly increased CXCR4-use compared to subtype B-infection [5]. In another study of 68 HIV-1 infected, pregnant, antiretroviral naïve women in Uganda, 9 (36%) of 25 subtype D viruses were DM tropic, while all the subtype A and A/D-recombinant viruses used CCR5 [6].

In Singapore, subtype B and CRF01_AE are the predominant HIV-1 circulating strains in a majority ethnic Chinese population [7]. In a recent survey, we described the presence of a novel circulating recombinant form, CRF51_01B, present in 12.8% of isolates [7,8]. Additionally, we documented the presence of inter-strain differences in clinical progression between subtype B and the recombinant strains [9]. This study aimed to determine inter-subtype differences in co-receptor usage in Singapore. Furthermore, we examined if stage of disease was a potential confounder in the subtype co-receptor relationship.

## Methods

Details of the study participants and laboratory protocols have been previously published [7]. From February 2008 to August 2009, this cross-sectional study enrolled 211 treatment-naïve HIV-1 patients presenting for care at the Singapore Communicable Disease Centre (CDC) outpatient HIV clinic. Blood from enrolled subjects was centrifuged to obtain plasma samples, stored at −80°C. Clinical and demographic information was obtained via chart review. This study was approved by the National Healthcare Group ethics committee, and written, informed consent were obtained from all subjects.

The QIAmp Viral RNA Mini Kit (Qiagen, Valencia, CA, USA) was used to extract viral RNA from plasma samples. As previously described, reverse-transcriptase polymerase chain reaction (RT-PCR), additional nested PCR, sample purification and Sanger sequencing were used to sequence portions of the gp120 (HXB2 6904 to 7628) [7]. In brief, the primers for env C2V5-F1, 5^′^-CTCCAGCTGGTTWTGCRATT-3′ (nt 6880–6899) and C2V5-R1 5′-GCCTGTACCGTCAGCGTTAT-3′ (nt 7827–7846) were used with the following conditions: 50°C for 30 minutes, 95°C for 15 minutes, 25 cycles at 94°C for 30 seconds, 55°C for 30 seconds, and 72°C for 1.5 minutes. Five microliters of the first-round PCR product was used in the second-round reaction in a 50 μL reaction mixture containing 0.8 mM of inter-nested primers C2-V5- F2 (inter) 5#-CAGCTGGTTWTGCGATTCTAA-3# (nt 6883–6903) and C2-V5-R2 (inter) 5#-RTYYCCTCCTCCAGGTCT- GA-3# (nt 7627–7646). Cycling conditions were 94°C for 15 minutes, followed by 35 cycles at 94°C for 30 seconds, 64°C for 30 minutes, and 72°C for 1.5 minutes. The current analysis was limited to subjects with interpretable gp120 sequences.

HIV-1 subtypes were assigned using the NCBI genotyping database and confirmed with phylogenetic analysis using reference sequences from the Los Alamos HIV Sequence Database, based on data from all genomic regions available. Nucleotide mixtures were considered when the second highest peak in the electropherogram was above 25%. V3 sequences harbouring nucleotide mixtures were translated into all possible amino acid permutations and subjected to the genotypic prediction test. Sequences with ≥8 nucleotide mixtures were excluded because of the high number of amino acid sequences generated. Genotypic co-receptor prediction was based on analysis of gp120 sequences using the online Geno2pheno tool [10]. Based on previous publications, two false-positive rates (FPR) cutoffs, 5.75% and 10%, were selected for co-receptor usage interpretation [2,11-13]. Samples below the FPR cutoff were classified as CXCR4, and those above or equal to the FPR cutoff were labeled as CCR5-using viruses. DM tropic viruses were classified as CXCR4-using virus for our analyses.

As previously described, evidence of recent seroconversion was defined as presence of a negative HIV test within 2 years prior to presentation for care [9]. Subject demographics, transmission risk factor, and median CD4 count at presentation, were analyzed by subtype. Genotypic co-receptor usage was analyzed by age, gender, ethnicity, transmission risk factor, median CD4 count at presentation, and subtype. Fisher’s exact test was used to compare categorical variables, and Wilcoxon rank-sum test and Median test were used for comparison of medians. To determine if stage of disease at presentation was a potential confounder of the relationship of subtype with co-receptor use, a stratified analysis limited to subjects with evidence of prior negative HIV test within 2 years before presentation for care was performed. For every parameter, only samples with complete information available were included in the analysis. All statistical analyses were performed using Stata 11 (StataCorp, College Station, TX, USA).

## Results

Of 211 enrolled in the study, 110 (52.1%) subjects had interpretable gp120 sequences, and were included for analysis. There were no significant differences in the age, gender, ethnicity, transmission risk factor or CD4 count at presentation.

The median age was 36.4 [28.5–45.0] years. Ninety-seven subjects (88.2%) were males. Eighty-eight (80%) subjects were ethnic Chinese, 10 (9.1%) subjects were Malays, and 12 (10.9%) of other ethnicity. Fifty (45.6%) reported heterosexual transmission risk, 46 (41.8%) homosexual transmission, 10 (9.1%) bisexual transmission, and 4 (3.7%) reporting other risk factors. The median CD4 count at presentation for care was 303 [128–425] cells/mm^3^.

Subtype distribution was as follows: 27 (24.6%) subtype B, 64 (58.2%) CRF01_AE, 10 (9.1%) CRF51_01B, and 9 (8.2%) other subtypes (2 CRF_02AG, 1 CRF07_BC, 2 CRF15_01B, 3 CRF33_01B, and 1 CRF34_01B). There was no statistically significant difference between subtypes, with regards to median age, gender, ethnicity or CD4 count at presentation for care (Table 1). A higher proportion of subtype B and CRF51_01B-infected subjects (51.9% and 50.0% respectively) reported homosexual transmission risk, compared to CRF01_AE-infected subjects and those with other subtypes (37.5% and 33.3% respectively) (*P* = 0.003).

**Table 1:** Baseline demographics and CD4 cell count, by subtype

**Patient characteristics**	**Subtype B (n=27), n (row %)**	**CRF01_AE (n=64), n (row %)**	**CRF51_01B (n=10), n (row %)**	**Others (n=9), n (row %)**	**P-value**
**Median age** (median, IQR)	28.9 [6.6–38.1]	37.2 [29.1–46.4]	39.8 [29.9–41.2]	36.7 [36.3–49.3]	0.092
**Male gender**	25 (92.6)	55 (85.9)	9 (90.0)	8 (88.9)	0.919
**Ethnicity**					
Chinese	18 (66.7)	54 (84.4)	9 (90.0)	7 (77.8)	0.227
Non-Chinese	9 (33.3)	10 (15.6)	1 (10.0)	2 (22.2)	
**Transmission risk**					
Heterosexual	8 (29.6)	37 (57.8)	1 (10.0)	4 (44.4)	0.003
MSM	14 (51.9)	24 (37.5)	5 (50.0)	3 (33.3)	
Others	5 (18.5)	3 (4.7)	4 (40.0)	2 (22.2)	
**CD4 count at presentation **(median, IQR)	416 [275–493]	263 [52–401]	167 [35–338]	248 [219–304]	0.092

Abbreviations: IQR, interquartile range; MSM, men who have sex with men.

^a ^Blood transfusion, intravenous drug use (IVDU), bisexual.

^b ^Other subtypes: CRF02_AG, CRF34_01B, CRF07_BC, CRF33_01B, CRF15_01B.

There was no statistically significant difference in median age, gender, ethnicity or transmission risk between subjects infected with CXCR4-using or CCR5-using virus (Table 2). Using a higher FPR=10%, subjects with CXCR4-using virus had a lower CD4 count at presentation for care (226 [32–402] cells/mm^3^) compared to subjects infected with CCR5-using virus (333 [200 to 462] cells/mm^3^) (*P* = 0.013). At a lower FPR of 5.75%, this association remained, with a median CD4 count of 219 (35–336) cells/mm^3^ in the CXCR4 population, and a median CD4 count of 333 [189–470] cells/mm^3^ in the CCR5 population. Among the same subtype, all 10 (100%) CRF51_01B-infected subjects and 26 out of 64 (40.6%) CRF01_AE-infected subjects had CXCR4-using virus, compared to 7 out of 27 (25.9%) subtype B and 1 out of 9 (11.1%) CRF33_01B-infected subjects at FPR=10% (*P* < 0.001). The association of subtype and co-receptor use remained significant when the FPR was changed to 5.75%. At FPR=5.75%, all 10 (100%) CRF51_01B-infected subjects and 20 out of 64 (31.3%) CRF01_AE-infected subjects had CXCR4-using virus, compared to 4 out of 27 (14.8%) subtype B and 1 out of 9 (11.1%) CRF33_01B-infected subjects (*P* < 0.001).

**Table 2:** Demographics and clinical characteristics, by co-receptor tropism

**FPR Criteria**	**FPR = 10**			**FPR = 5.75**		
**Patient characteristics**	**CXCR4 (n=44), n (row %)**	**R5 (n=66), n (row %)**	**P-value**	**CXCR4 (n=35), n (row %)**	**R5 (n=75), n (row %)**	**P-value**
**Median age** (median, IQR)	36.9 [28.8–45.4]	36.4 [27.6–44.6]	0.665	39.7 [29.4–45.4]	35.6 [27.6–45.0]	0.539
**Male gender**	38 (39.1)	59 (61.0)	0.765	30 (30.9)	67 (69.1)	0.752
**Ethnicity**						
Chinese	34 (77.3)	54 (81.8)	0.630	27 (77.1)	61 (81.3)	0.617
Non-Chinese	10 (22.7)	12 (18.2)		8 (22.9)	14 (18.7)	
**Transmission risk**						
Heterosexual	20 (45.5)	30 (45.5)	0.945	15 (42.9)	36 (48.0)	0.502
MSM	23 (52.3)	33 (50.0)		14 (40.0)	32 (42.7)	
Others^a^	1 (3.0)	3 (4.5)		6 (17.1)	7 (9.3)	
**CD4 count at presentation **(median, IQR)	226 [32–402]	333 [200–462]	0.013	219 [35–336]	333 [189–470]	0.025
**Evidence of recent seroconversion**(n=25)						
Subtype B	2 (8.0)	4 (16.0)	0.011	1 (4.0)	5 (20.0)	0.007
CRF01_AE	3 (12.0)	10 (40.0)		3 (12.0)	10 (40.0)	
CRF51_01B	5 (2.0)	0 (0.0)		5 (20.0)	0 (0.0)	
Others^c^	0 (0.0)	1 (4.0)		0 (0.0)	1 (4.0)	

Abbreviations: IQR, interquartile range; MSM, men who have sex with men; FPR, false-positive rate.

^a ^Blood transfusion, intravenous drug use (IVDU), bisexual.

^b ^Other subtypes: CRF02_AG, CRF34_01B, CRF07_BC, CRF15_01B.

^c ^Other subtypes: CRF33_01B.

To examine the influence of stage of disease, an analysis limited to individuals with evidence of recent seroconversion (n=25) was performed. Nine (36.0%) of 25 recently-infected individuals had CXCR4-using virus, compared to 26 (30.6%) of 85 individuals without evidence of recent infection (*P =* 0.631). Excluding the 10 CRF51_01B-infected individuals from analysis, 4 (20.0%) of 20 recently-infected individuals had CXCR4-using virus compared to 21 (26.3%) of 80 individuals without evidence of recent infection (*P =* 0.774). At FPR=5.75%, all CRF51_01B and 3 out of 13 (23.1%) CRF01_AE-infected subjects had CXCR4-using virus compared to 1 out of 6 (16.7%) of the subtype B and none from the CRF33_01B-infected subjects (*P* = 0.001). At FPR=10%, all CRF51_01B-infected subjects had CXCR4-using virus compared to 0 to 33% of the other subtypes with CXCR4-usage (*P* = 0.011). We repeated the analysis, limiting the subjects to those meeting the definition of recent seroconversion as a maximum interval of 1 year between the last negative HIV-1 test and first positive HIV-1 test. The inferences remained unchanged with a significantly higher proportion of CRF51_01B-infected subjects having CXCR4-using virus (data not shown).

## Discussion

Our study found that CRF51_01B and CRF01_AE HIV-1 virus had higher prevalence of CXCR4-usage compared to subtype B. Interestingly, all CRF51_01B isolates, including those from subjects with evidence of recent seroconversion, were CXCR4-using. Limiting the analysis to subjects with evidence of recent seroconversion, the difference in proportion of CXCR4-using virus among subtype B and CRF01_AE-infected subjects was less apparent, possibly due to smaller numbers of subjects.

The high prevalence of CXCR4-use among CRF01_AE-infected subjects could be a factor in the increased rates of CD4 T-cell decline previously observed among CRF01_AE compared to subtype B-infected subjects [9]. The high prevalence of CXCR4-use among CRF51_01B-infected subjects suggests that CRF51_01B-infected patients could experience faster immunologic decline compared to subtype B-infected patients; a hypothesis supported by preliminary analyses documenting higher proportion of AIDS at presentation, and greater rate of CD4 T-cell decline among CRF51_01B-infected subjects compared to subjects infected with subtype B [14]. The virion of CRF51_01B comprises a recombinant structure of CRF01_AE and subtype B [7,8]. Specifically, portions of CRF51_01B harbour CRF01_AE in the gp120 and subtype B in the protease and gp41 regions. Given the unique patterns in inter-strain recombination, future comparative analysis on the viral genetic elements could potentially lead to the identification of key determinants that account for the distinct biological phenotype (including tropism and co-receptor usage) implicated in this study.

For clinical practice, our current findings highlight the need for co-receptor determination prior to prescribing CCR5-antagonist to individuals infected with CRF01_AE or CRF51_01B [2]. As noted in a recent meta-analysis and consensus statement on the clinical management of HIV-1 tropism testing, genotypic analysis of the HIV third hypervariable loop (V3) is the most practical modality of testing. The laboratory turnaround time for phenotypic testing precludes widespread clinical use of this method. In the above noted meta-analysis and consensus statement, the Geno2pheno interpretation system was recommended as the best-validated interpretation algorithm, while acknowledging the dearth of data in non-B subtypes. Accordingly, there is need for better data and potentially improved algorithms tailored for non-B HIV-1 subtypes.

V3-based genotyping algorithms are generally less sensitive in identifying X4 variants for non-B subtypes because they were built using datasets of genotype-phenotype correlations from subtype B viruses [15-17]. According to a study evaluating the reliability of several V3-based genotypic predictors, the sensitivity to identify X4 variants was 90% for B subtypes and 61% for non-B subtypes using Geno2pheno compared with the phenotypic assay [15]. Seclen *et al.* determined a sensitivity of 58% and specificity of 79% for non-B subtypes, compared with a sensitivity of 94% and specificity of 51% for subtype B, using the Geno2pheno tool [17]. The inadequacy of current genotypic tools for predicting CXCR4-using viruses among non-B subtypes thus underscores the need to improve the information on the correlation between phenotypic and genotypic methods for viral tropism determination in patients infected with non-B HIV-1.

Some limitations of this study and the use of genotypic co-receptor prediction assay should be acknowledged. Due to the genetic variations in the diverse sample population, it was technically challenging to optimize the cycling conditions that could be universally applied for the DNA cycle sequencing of all HIV subtypes. This has likely resulted in the lower proportion (51%) of interpretable gp120 sequences reported in the study herein. Secondly, our study lacks phenotypic confirmation of co-receptor status. Genotypic co-receptor determination is best validated for subtypes B and C, and little data exists specifically on its utility for CRF01_AE isolates [2]. A prior study with a cohort of 103 patients harbouring various subtypes demonstrated a good correlation of 88% sensitivity and 87% specificity against phenotypic assessment of HIV-1 co-receptor usage [18]. Another study examining the accuracy of Geno2pheno prediction at 10% FPR among 23 subtype B and 79 non-B isolates demonstrated a 97% sensitivity, 93% specificity, compared to phenotypic co-receptor determination [19]. However, poor genotype-phenotype correlations have also been reported in some studies, especially for the detection of X4 variants. Retrospective analysis of a cohort of treatment-experienced patients enrolled in the framework of two phase III clinical trials MOTIVATE 1 and 2 showed that sensitivity of Geno2pheno to detect X4 variants was 63% compared with the Trofile™ phenotypic assay, even though the genotypic test was able to distinguish between responders and non-responders to maraviroc [11].

## Conclusion

In conclusion, the study herein showed that HIV-1 subtype CRF51_01B and CRF01_AE-infected subjects have higher prevalence of CXCR4-usage compared to subtype B. Larger studies involving phenotypic co-receptor determination would strengthen these findings. Further studies examining the clinical implications of these findings would help optimise the usage of CCR5-antagonists in treatment, and further our understanding of HIV biology.

## Competing interests

The authors declare that they have no competing interests.

## Authors’ contributions

KYN and OTN conceived of the study and carried out its design. KKC, PK and JKY performed the assays. KYN, WXK and OTN drafted the manuscript. LL, AC, MTT, TCQ, OL and YSL provided guidance on data analysis and contributed to writing. All authors read and approved the final manuscript.

## Pre-publication history

The pre-publication history for this paper can be accessed here:

http://www.biomedcentral.com/1471-2334/13/90/prepub
